# A novel risk-predicted nomogram for sepsis associated-acute kidney injury among critically ill patients

**DOI:** 10.1186/s12882-021-02379-x

**Published:** 2021-05-10

**Authors:** Shanglin Yang, Tingting Su, Lina Huang, Lu-Huai Feng, Tianbao Liao

**Affiliations:** 1 Department of Academic Affairs Office, YouJiang Medical University for Nationalities, Baise, China; 2 Department of ECG Diagnostics, The People’s Hospital of Guangxi Zhuang Autonomous Region, Nanning, China; 3 Department of Comprehensive Internal Medicine, The Affiliated Tumor Hospital of Guangxi Medical University, Nanning, China; 4 Department of President’s Office, YouJiang Medical University for Nationalities, Baise, China

**Keywords:** Sepsis, Acute kidney injury, Prediction, Nomogram, Intensive care unit

## Abstract

**Background:**

Acute kidney injury (AKI) is a prevalent and severe complication of sepsis contributing to high morbidity and mortality among critically ill patients. In this retrospective study, we develop a novel risk-predicted nomogram of sepsis associated-AKI (SA-AKI).

**Methods:**

A total of 2,871 patients from the Medical Information Mart for Intensive Care III (MIMIC-III) critical care database were randomly assigned to primary (2,012 patients) and validation (859 patients) cohorts. A risk-predicted nomogram for SA-AKI was developed through multivariate logistic regression analysis in the primary cohort while the nomogram was evaluated in the validation cohort. Nomogram discrimination and calibration were assessed using C-index and calibration curves in the primary and external validation cohorts. The clinical utility of the final nomogram was evaluated using decision curve analysis.

**Results:**

Risk predictors included in the prediction nomogram included length of stay in intensive care unit (LOS in ICU), baseline serum creatinine (SCr), glucose, anemia, and vasoactive drugs. Nomogram revealed moderate discrimination and calibration in estimating the risk of SA-AKI, with an unadjusted C-index of 0.752, 95 %Cl (0.730–0.774), and a bootstrap-corrected C index of 0.749. Application of the nomogram in the validation cohort provided moderate discrimination (C-index, 0.757 [95 % CI, 0.724–0.790]) and good calibration. Besides, the decision curve analysis (DCA) confirmed the clinical usefulness of the nomogram.

**Conclusions:**

This study developed and validated an AKI risk prediction nomogram applied to critically ill patients with sepsis, which may help identify reasonable risk judgments and treatment strategies to a certain extent. Nevertheless, further verification using external data is essential to enhance its applicability in clinical practice.

## Background

Sepsis is a major global cause of high morbidity and mortality for critically ill patients [1] with a continuously increasing incidence [2]. Besides, sepsis is an enormous burden, accounting for ∼850,000 emergency visits per year and up to 381,000 annual related deaths in the USA [2, 3]. Sepsis can lead to various complications. For instance, SA-AKI is a common and severe complication of sepsis that meets consensus criteria for both sepsis and AKI [4], indicating multiple organ dysfunction and significant poor clinical outcomes [5, 6]. Noteworthy, sepsis is associated with up to 50 % of AKI [7], and up to 60 % of patients with sepsis have AKI [8]. Among critically ill patients with SA-AKI, the mortality rates range from 38.2 to 70.2 % [8, 9]. Nevertheless, so far, no single effective therapy has been reported to change the outcome of SA-AKI [10]. Notably, early diagnosis and treatment improve the long-term outcome of patients. Therefore, early identification of high-risk patients is important for AKI prevention [11].

SA-AKI is diagnosed based on specific, context-dependent, and imperfect definitions, besides, an increase in serum creatinine or a decline in urine output remains its key diagnostic criteria [12]. Accumulating knowledge has highlighted the clinical risk factors, pathobiology, response to treatment, and elements of renal recovery thereby improving the prevention, detection, and treatment of SA-AKI [4]. Nonetheless, the pathogenesis of SA-AKI remains completely unclear and its risk factors emerge from various sources, making it a major clinical challenge in early detection [13, 14]. Despite many novel biomarkers related to the early diagnosis and prognosis of SA-AKI being reported, few are applied in clinical practice [15]. Recent studies have used the patient and disease characteristics of AKI to identify patients with an increased risk, however, most of them have not integrated these with clinical prediction models [16–19]. Other studies have developed risk assessment models for AKI in patients based on patient and disease characteristics alone [20, 21], yet few have been developed for SA-AKI. As such, there is an urgent need for a more modern framework for rapid clinical diagnosis of SA-AKI.

A nomogram is a reliable tool that predicts and quantifies risk for a clinical event by creating a visualized graph of the predictive model based on relevant factors [22, 23]. Herein, we identified a combination of routinely available clinical variables for a highly precise prediction of SA-AKI in critically ill patients.

## Methods

### Database

Retrospectively, data were extracted from the MIMIC-III database (version 1.4), a large US-based, publicly available critical care database [24]. The MIMIC-III (v1.4) database included unidentified health-related data of 61,532 ICU stays at the Beth Israel Deaconess Medical Center between June 2001 and October 2012. The establishment of MIMIC-III (v1.4) was approved by the institutional review boards of the Beth Israel Deaconess Medical Center (Boston, MA) and Massachusetts Institute of Technology (Cambridge, MA), thus, this study was granted a waiver of informed consent. One author (L-H F) completed the online training course of the National Institutes of Health (certification number 35,897,462) to access the MIMIC-III (v1.4).

### Participants

Adult patients (≥ 18 years old) diagnosed with sepsis identified from the International Classification of Diseases 9th Edition (ICD-9) code were selected from the MIMIC-III v1.4 database. Patients with AKI before admission to the ICU were excluded. For patients with more than one ICU stay, only the first ICU admission of each patient was analyzed.

A pre-seeded random number (123) generator in R software (version 3.6.2) was used to determine the grouping. Finally, the patients were randomly divided into primary (*n* = 2012) and validation (*n* = 859) cohorts based on the ratio of 7:3.

### Data extraction

Data extraction was performed using PostgreSQL tools (V.1.13.1). The following information was extracted directly or calculated using data from the database: Age, gender, body mass index (BMI), Systemic inflammatory score (SIRS), laboratory variables, chronic medical conditions, comorbidities, length of stay in the intensive care unit, the time of AKI, administration of drugs. Laboratory variables including hemoglobin, platelet counts, glucose, serum creatinine, and albumin were measured during the first 24 h in the ICU. Chronic medical conditions included chronic obstructive pulmonary disease (COPD), chronic kidney disease (CKD), diabetes, coronary disease, malignant, chronic liver disease, and hypertension. Comorbidities included acute pancreatitis, lactic acidosis, heart failure, and hypotension. Notably, comorbidities and chronic medical conditions were collected based on the recorded ICD-9 codes in the MIMIC-III database. The drugs administrated to patients included vasoactive drugs, diuretic, aminoglycosides, lactated Ring, and human albumin. Variables associated with the risk of SA-AKI were assessed a priori based on scientific knowledge, clinical importance, and predictors identified in previously published articles [9, 14, 25].

### Definitions and outcomes

AKI during ICU stay was the primary outcome. AKI was defined following the Kidney Disease Improving Global Outcomes (KDGIO) criteria [26]. Considering that the patients might have used diuretics, AKI was defined only based on the change of serum creatinine value. Vasoactive drugs, diuretic, and aminoglycosides were defined as any vasoactive drugs, diuretic and aminoglycosides use during ICU stay for any reason. The severity of anemia was established following the reference standard of the World Health Organization (WHO) [27]. WHO international BMI cut-off points were used for BMI group categorization [28]: underweight (BMI < 18.5), normal weight (BMI 18.5 to 24.9), overweight (BMI 25 to 29.9), and obesity (BMI > 30).

### Statistical analysis

Continuous variables were presented as interquartile ranges (*M* (*P*_25_, *P*_75_)) unless indicated otherwise while categorical variables were presented as frequency and proportion of patients in each category. In the primary cohort, the assumption of linearity in the logistic for the continuous variable was assessed and univariate logistic analyses were used to analyze the relationships of relevant variables with SA-AKI. All variables with P < 0.05 in the univariate logistic analyses were further assessed by multivariable logistic regression using backward stepwise selection, where the variable with the largest p-value was eliminated at each step until all remaining variables had significant *p* < 0.05. Multicollinearity was evaluated using variance inflation factors and there was no evidence of Multicollinearity. Nomograms predicting the risk of SA-AKI were determined using the independently selected significant variables. While ensuring the stability of prediction performance, a few features were removed to simplify the nomogram [23].

The performance of the nomograms was evaluated using the C-index [29] and calibration curves. The discriminative ability of the nomograms was evaluated by C-index, where a C-index of 0.5 indicated the absence of discrimination, whereas a C-index of 1.0 suggested perfect discrimination. Calibration was assessed using calibration curves, graphic representation of the relationship between the frequency of observations and the probability of prediction, with a 1000-bootstrapped sample of the primary cohort. The final nomogram was verified in the validation cohort to assess the stability and generality of the nomogram. Moreover, the clinical utility of the final nomogram was evaluated using decision curve analysis by quantifying the net benefit at different threshold probabilities. The net benefit was calculated by subtracting the proportion of false positives from the proportion of true positives and weighing by the relative harm of foregoing treatment compared to the negative consequences of an unnecessary treatment [30].

For missing data, median imputation was used if the numbers were small (< 5 %), while multiple imputations were used if the numbers with missing data were large. All statistical analyses were performed using the R software (version 3.6.0). All tests were two-sided, with a significance level of 5 %.

## Results

### Characteristics of patients with SA‑AKI

In total, 2,871 patients with sepsis were recruited and 1,137 patients (39.6 %) positively tested for SA‑AKI. The mean age of patients was 67 years, and a majority (55.1 %) were male. Patients were randomly assigned to primary (2,012 patients) or validation (859 patients) cohorts. Table 1 shows the characteristics of the patient in each cohort. The baseline clinical characteristics were similar between the two cohorts, with SA‑AKI proportions of 39.2 and 40.6 % in the primary and validation cohorts, respectively.

**Table 1:** Characteristics of Patients in the Primary and Validation Cohorts after randomization

Variable	Primary Cohort	Validation Cohort
Age (years)	69 (56,81)	68 ( 55,81)
Male, n (%)	1112 (55.27)	470 ( 54.71)
BMI, n (%)		
Normal	624 ( 31.01)	254 (29.57 )
Overweight	396 ( 19.68)	203 ( 23.63 )
Obesity	923 (45.87)	366 (42.61)
Underweight	69 (3.43 )	36 ( 4.19 )
LOS in ICU (days)	3.7 ( 1.9, 8.9 )	2.9 ( 1.9, 9.9 )
SIRS	3 ( 3, 4)	3 ( 3, 4)
Anemia, n (%)		
Normal	491 ( 24.40 )	176 ( 20.49 )
Mild anemia	1090 ( 54.17 )	460 ( 53.55 )
Moderate anemia	428 ( 21.27 )	222 (25.84 )
Severe anemia	3 ( 0.15 )	1 ( 0.12 )
Glucose ( mmol/L)	7.0 ( 4.3, 11.9)	7.0 ( 4.2, 11.4 )
Basline SCr ( umol/L)	124 ( 80,194)	124 ( 80,203)
Albumin, n (%)		
< 30 g/L	932 ( 46.32 )	420 ( 48.89 )
>= 30 g/L	1080 ( 53.68 )	439 ( 51.11 )
Chronic medical conditions, n (%)		
COPD	44 ( 2.19 )	15 ( 1.75 )
CKD	338 ( 16.8 )	165 ( 19.21 )
Chronic liver disease	105 ( 5.22 )	59 ( 6.87 )
Diabetes	633 ( 31.46 )	254 ( 29.57 )
Coronary disease	334 ( 16.60 )	121 ( 14.09 )
Malignant	439 ( 21.82 )	176 ( 20.49 )
Hypertension	798 ( 39.66 )	311 ( 36.20 )
Comorbidity, n (%)		
Acute pancreatitis	115 ( 5.72 )	39 ( 4.54 )
Lactic acidosis	469 ( 23.31 )	209 ( 24.33 )
Heart failure	631 ( 31.36 )	253 ( 29.45 )
Hypotension	110 ( 5.47 )	54 ( 6.29 )
Medication, n (%)		
Vasoactive drugs	891 ( 44.28 )	388 ( 45.17 )
Diuretic	760 ( 37.77 )	301 ( 35.04 )
Aminoglycosides	1516 ( 75.35 )	645 ( 75.09 )
Lactated Ring	118 ( 5.86 )	52 ( 6.05 )
Human albumin	222 ( 11.03 )	91 ( 10.59 )

*BMI* body mass index; *SCr* Serum creatinine; *LOS in ICU* length of stay in intensive care unit; *SIRS* Systemic inflammatory score; *COPD* chronic obstructive pulmonary disease; *CKD* chronic kidney disease

### Model specifications and predictors of SA-AKI

Established risk factors, AKI, and demographic characteristics of clinical importance were selected as candidate variables for the prediction model. Variables relevant to SA-AKI in the primary cohort were subjected to univariate and multivariate logistic regression analyses, and the result is illustrated in Tables 2 and 3. Final statistical analysis identified 12 predictors for SA-AKI, including BMI, LOS in ICU, baseline SCr, glucose, anemia incidence, CKD, heart failure, coronary disease, chronic liver disease, and the use of human albumin or vasoactive drugs.

**Table 2:** Univariate Logistic Regression Analysis of Factors relating to SAAKI in Primary Cohort

Variable	OR	95 % CI	*P*-Value
Age	0.85	0.74–0.96	0.119
Gender	1.09	0.91–1.30	0.371
BMI	1.43	1.18–1.74	< 0.001
LOS in ICU	1.81	1.64–2.00	< 0.001
SIRS	1.34	1.19–1.50	< 0.001
Basline SCr	1.26	1.17–1.37	< 0.001
Anemia	3.38	2.27–5.04	< 0.001
Glucose	1.35	1.24–1.46	< 0.001
Albumin	1.74	1.45–2.08	< 0.001
Chronic medical conditions			
COPD	1.11	0.61–2.03	0.730
CKD	1.96	1.55–2.47	< 0.001
Diabetes	1.44	1.19–1.75	< 0.001
Chronic liver disease	2.16	1.49–3.13	< 0.001
Coronary disease	1.46	1.15–1.86	0.002
Malignant	0.94	0.76–1.18	0.610
Hypertension	0.92	0.77–1.10	0.365
Comorbidity			
Acute pancreatitis	1.49	1.00–2.21	0.048
Lactic acidosis	1.50	1.22–1.85	< 0.001
Heart failure	1.66	1.37–2.01	< 0.001
Hypotension	1.05	0.71–1.55	0.799
Medication			
Vasoactive drugs	2.27	1.89–2.72	< 0.001
Diuretic	0.88	0.74–1.06	0.191
Aminoglycosides	1.56	1.26–1.93	< 0.001
Lactated Ring	0.90	0.62–1.30	0.569
Human albumin	2.78	2.08–3.71	< 0.001

*BMI* body mass index; *SCr* Serum creatinine; *LOS in ICU* length of stay in intensive care unit; *SIRS* Systemic inflammatory score; *COPD* chronic obstructive pulmonary disease ;*CKD* chronic kidney disease

**Table 3:** Results of the forward stepwise logistic regression analysis of SA-AKI in Primary Cohort

Variable	OR	95 % CI	*P*-Value
BMI	1.40	1.13–1.75	0.003
LOS in ICU	1.65	1.50–1.82	< 0.001
SIRS	1.11	0.98–1.26	0.114
Basline SCr	1.19	1.08–1.30	< 0.001
Anemia	2.24	1.42–3.55	< 0.001
Glucose	1.21	1.11–1.33	< 0.001
Albumin	1.33	1.08–1.63	0.007
Chronic medical conditions			
CKD	1.74	1.31–2.30	< 0.001
Chronic liver disease	1.75	1.13–2.71	0.012
Diabetes	1.04	0.83–1.31	0.752
Coronary disease	1.44	1.09–1.91	0.012
Comorbidity			
Acute pancreatitis	0.88	0.56–1.39	0.590
Lactic acidosis	1.06	0.84–1.35	0.613
Heart failure	1.29	1.03–1.62	0.025
Medication			
Vasoactive drugs	2.15	1.74–2.66	< 0.001
Aminoglycosides	1.15	0.90–1.48	0.267
Human albumin	2.55	1.83–3.56	< 0.001

*BMI* body mass index; *SCr* Serum creatinine; *LOS in ICU* length of stay in intensive care unit; *SIRS* Systemic inflammatory score; *COPD* chronic obstructive pulmonary disease ;*CKD* chronic kidney disease

### Nomograms and model performance in the primary cohort

**Fig. 1:**
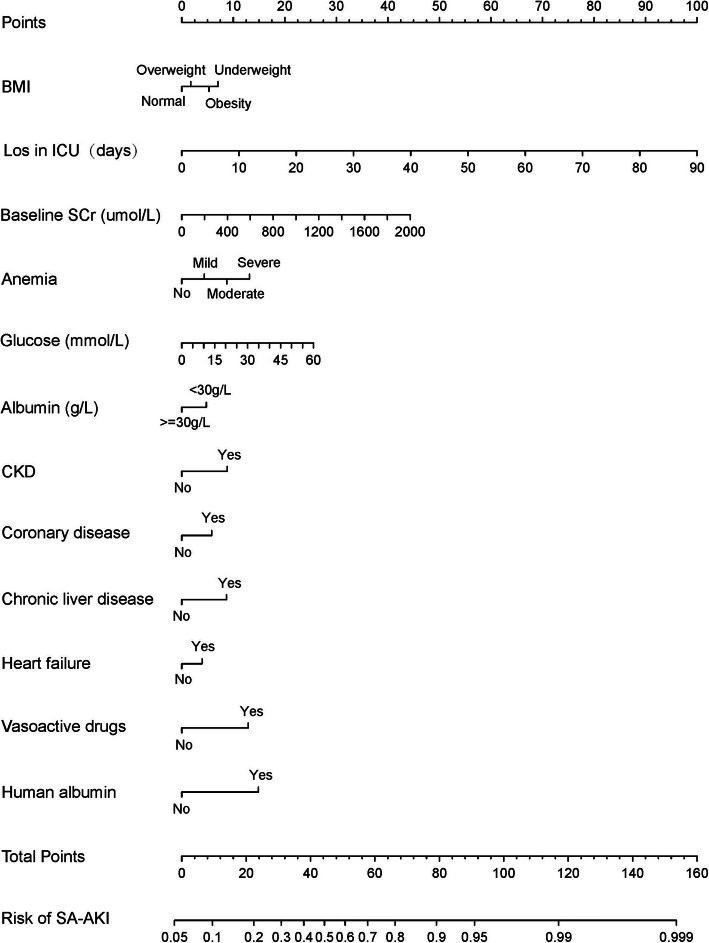
Nomogram 1 developed based on the primary cohort with the incorporation of 12 predictors

**Fig. 2:**
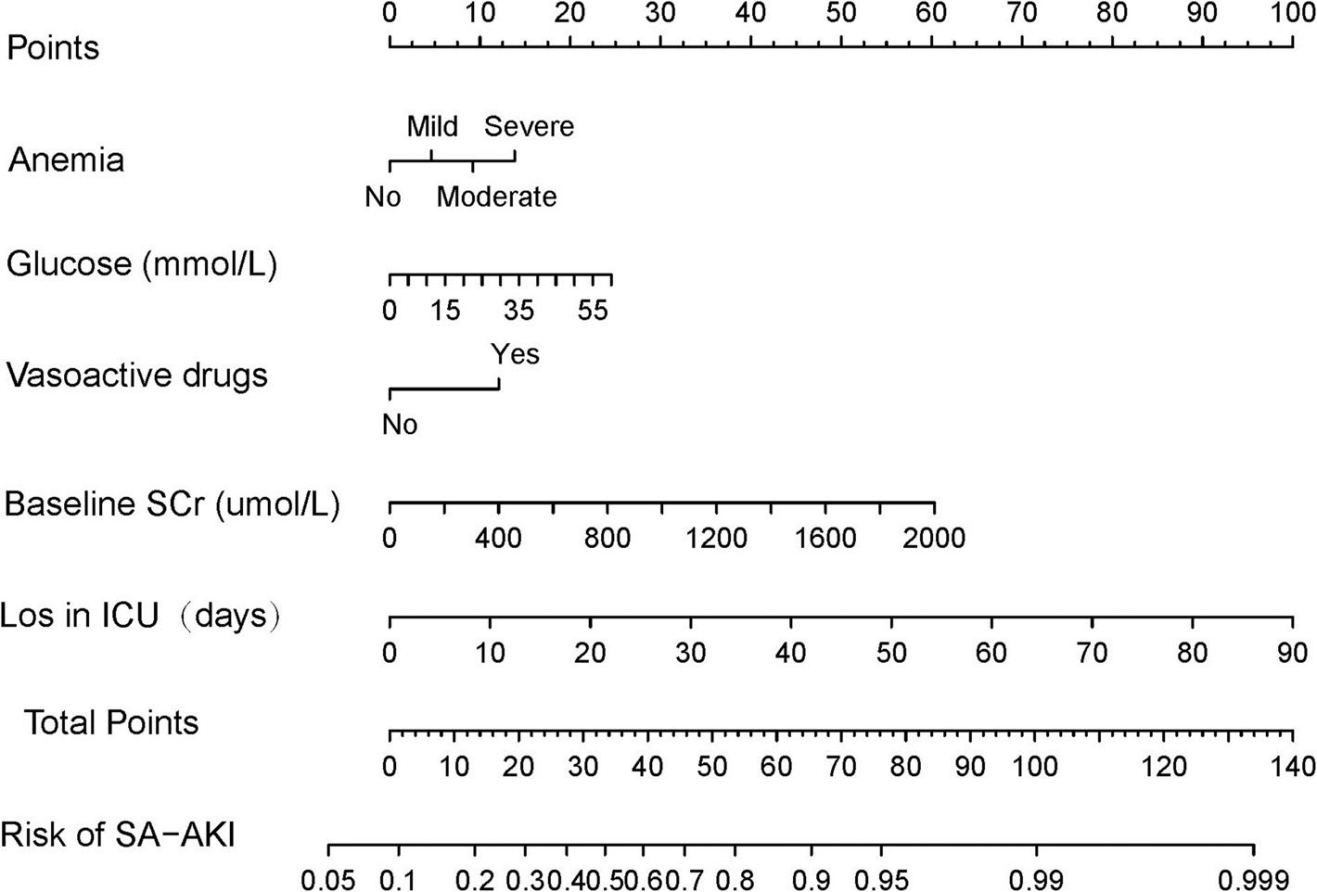
Nomogram 2 developed based on the primary cohort with the incorporation of LOS in ICU, baseline SCr, glucose, anemia, and vasoactive drugs

Nomograms for SA-AKI incorporating significant predictive factors from the multivariate analysis were established (Figs. 1 and 2). Nomogram 1 included 12 significant predictors for SA-AKI prediction (Fig. 1), depicting moderate discrimination in prediction with an unadjusted C-Index of 0.773 (95 %Cl, 0.752–0.794). However, nomogram 1, combining 12 predictors was cumbersome. Besides, BMI, albumin, CKD, heart failure, coronary disease, chronic liver disease, and human albumin predictors demonstrated insignificant influence on the point in nomogram 1. For effective visualization, a few predictors were reduced to simplify the nomogram. Nomogram 2 (Fig. 2) including factors with LOS in ICU, baseline SCr, glucose, anemia, and vasoactive drugs maintained similar discrimination (C-index 0.752, 95 %Cl [0.730–0.774]).

**Fig. 3:**
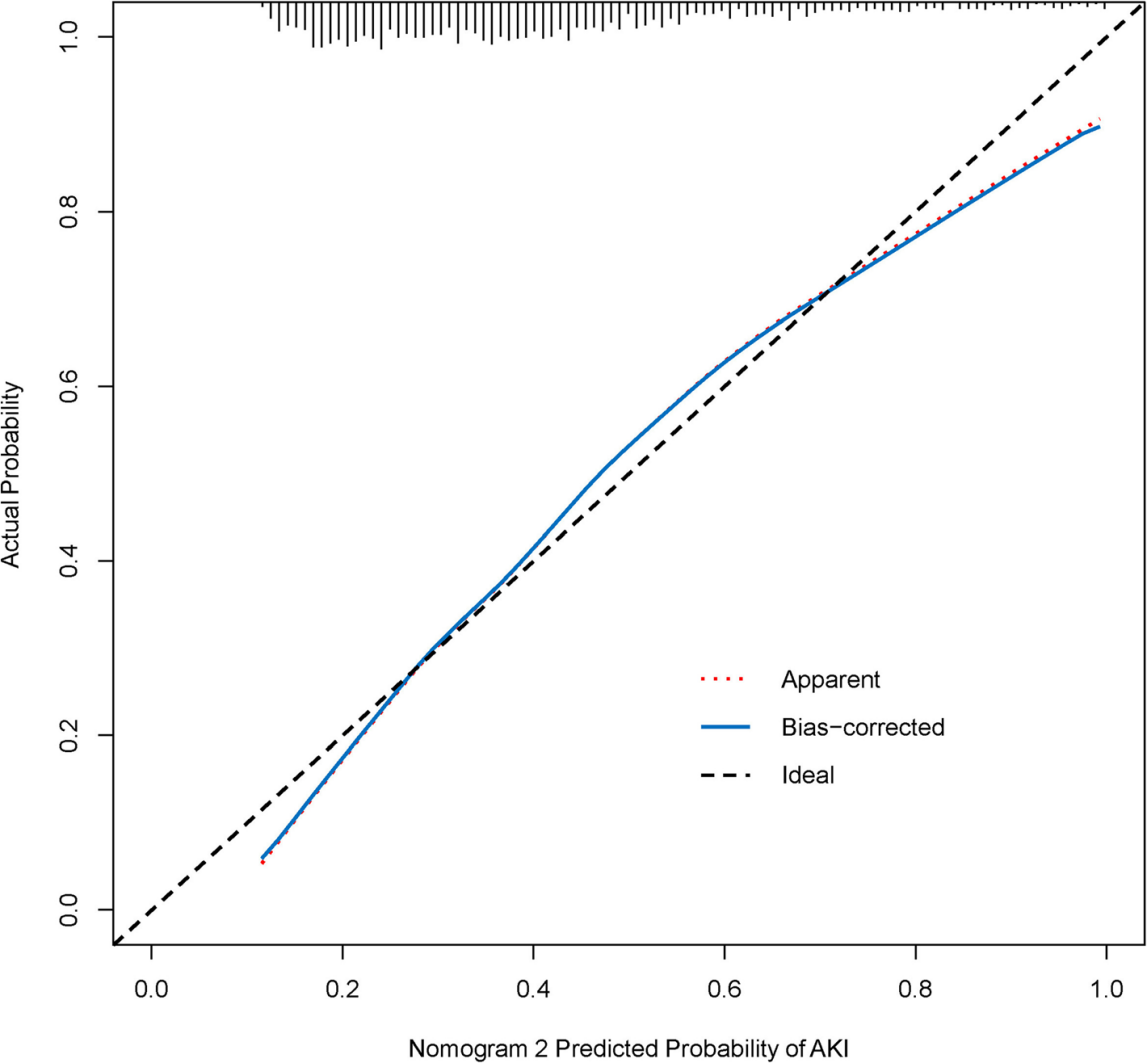
Calibration curves for nomogram 2 in the primary cohort. The blue dotted line represents the entire cohort (*n *= 2,012), and the red solid line depicts the result after bias-correction by bootstrapping (1000 repetitions), indicating the performance of nomogram 2

Therefore, nomogram 2 was identified as the effective predictive model. To use nomogram 2, a vertical line should be delineated to the point raw to assign point values for each predictor. Each patient obtains a total point by plus the points of five predictors in nomogram 2. For instance, a patient with mild anemia, the glucose of 10 mmol/L, is using vasoactive drugs, baseline SCr of 200 umol/L and 10 days Los in ICU, total point taken from nomogram 2 of the patient is 38 (5 + 4 + 12 + 6 + 11 = 38), and the risk of SA-AKI probability is 60 %. The weights for each feature are list in Table 4 for calculation without a nomogram. The bootstrap-corrected C-index for the prediction nomogram 2 was 0.749 and the calibration plot for the probability of SA-AKI revealed a good correlation between nomogram 2 prediction and actual observation (Fig. 3), indicating moderate discrimination by our final model.

**Table 4:** the points for predictors

Predictor	points
Anemia	
Normal	0
Mild anemia	5
Moderate anemia	9
Severe anemia	14
Glucose ( mmol/L)	
5	2
10	4
15	6
20	8
25	10
30	12
35	14
40	16
45	18
50	20
55	22
60	25
Vasoactive drugs	
No	0
Yes	12
baseline SCr (umol/L)	
200	6
400	12
600	18
800	24
1000	30
1200	36
1400	42
1600	48
1800	54
2000	60
LOS in ICU (days)	
10	11
20	22
30	33
40	44
50	56
60	67
70	78
80	89
90	100
Total points	Risk of SA-AKI
1	10.0 %
13	20.0 %
21	30.0 %
27	40.0 %
33	50.0 %
39	60.0 %
46	70.0 %
54	80.0 %
65	90.0 %
76	95.0 %
100	99.0 %
134	99.9 %

### External validation of the nomogram 2 in the validation cohort

**Fig. 4:**
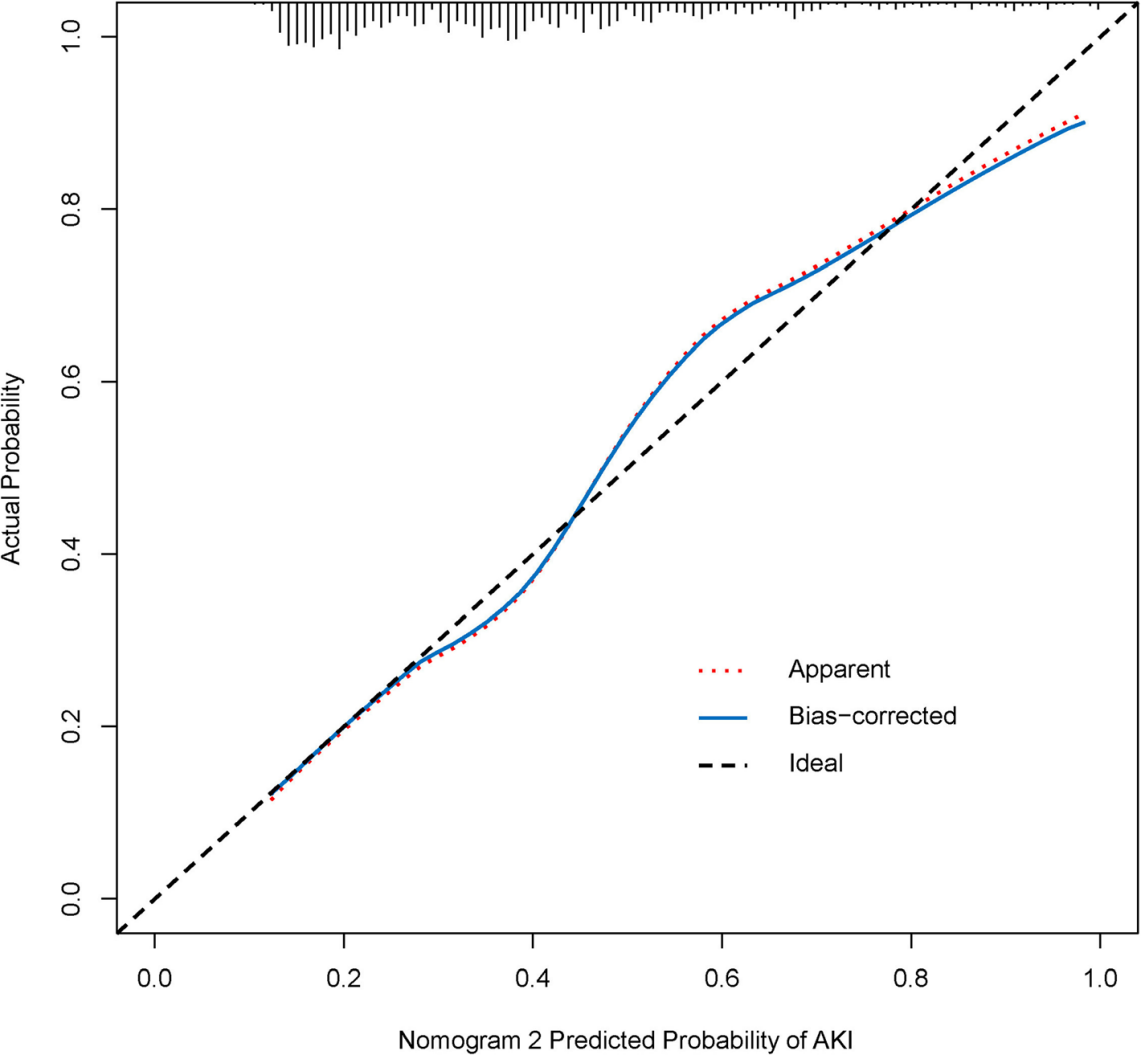
Calibration curves for nomogram 2 in the validation cohort. The blue dotted line represents the entire cohort (n = 859), and the red solid line is the result after bias correction by bootstrapping (1000 repetitions), indicating the performance of nomogram 2

In the validation cohort, nomogram 2 displayed a C-index of 0.757 (95 % CI 0.724–0.790) for estimation of SA-AKI risk. Also, there was a good calibration curve for risk estimation (Fig. 4).

### Clinical use of nomogram 2

**Fig. 5:**
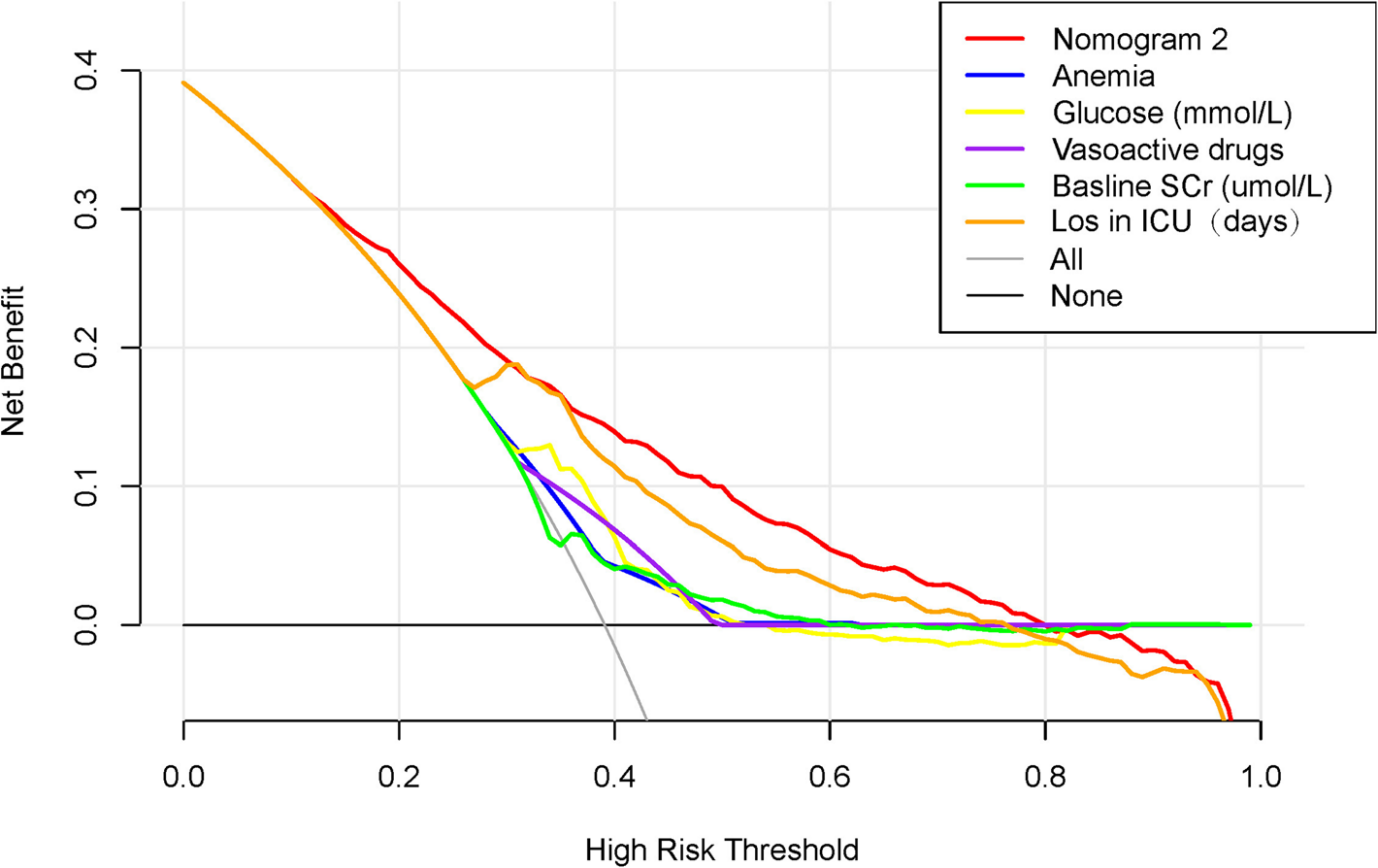
Decision curve analysis for nomogram 2, LOS in ICU, baseline SCr, glucose, anemia, and vasoactive drugs

The decision curve analysis (DCA) for nomogram 2 and the individual predictor is illustrated in Fig. 5. The DCA revealed that nomogram 2 would effectively predict SA-AKI if the threshold probability of SA-AKI is between 15 and 80 %. Within this range, the predictive effect of the nomogram is better than that of a single predictor, respectively.

## Discussion

Sepsis Associated Acute Kidney Injury (SA-AKI) is a frequent complication among critically ill patients causing alarming mortality and morbidity. SA-AKI has a higher risk of in-hospital death and longer hospital stay than AKI caused by other factors [31]. However, crude markers of renal function including SCr and Urine output are not effective in early diagnosis. This study developed and verified a diagnostic nomogram for predicting SA-AKI in critically ill patients. The nomogram incorporates five items, including LOS in ICU, baseline SCr, glucose, anemia, and vasoactive drugs which can be used to diagnose critically ill patients, enabling individualized decisions on the surveillance and treatment. Our findings are valuable since the nomogram was established based on a large database of critically ill patients. Additionally, in contrast with previously proposed models based on the clinical risk factors [25], the current nomogram has stable performance with ease of visualization.

Inflammation, oxidative stress, microvascular endothelial dysfunction, and renal tubular epithelial cell injury are the potential mechanisms of SA-AKI, however, its pathogenesis remains elusive [9]. The occurrence and development of these mechanisms [4, 9, 20] are potentially attributed to the variables in our model which might promote or balance each other and influence the process of SA-AKI. Therefore, predicting the occurrence of SA-AKI via these clinical variables is of importance.

Our study considered a wide array of clinical risk predictors previously linked to SA-AKI [4, 9, 14]. Specifically, in the medical treatment, we noted no relationship between diuretic, aminoglycosides, and SA-AKI. In contrast, data on SA-AKI demonstrated that diuretics might not be conducive to the prevention of AKI [4], and aminoglycosides are harmful to renal function. Predictors associated with AKI extensively vary, but rarely for sepsis and limited studies have integrated them with clinical prediction models. Recently, Zhou et al. analyzed 2,617 patients with sepsis and constructed a comprehensive risk score comprising 16 factors to predict SA-AKI [25], and lacked analysis of drug use (For example, vasoactive drugs), which might be unspecific for clinical use. The multivariable model including all contrast-enhanced ultrasonography variables created by Liu et al. was also complex and unsuitable for quick risk assessment [32]. This work differs from the previous simple multi-factor analysis [33] in that we removed predictors with little effect on the points in the nomogram to simplify the model for clinical application. Furthermore, none of the existing risk models was estimated by the DCA for their clinical utility. To our knowledge, this is the first risk model to consider previously proposed risk factors to develop a nomogram for prediction the prognosis of SA-AKI.

Since the treatment strategies are potentially heterogeneous, accurate AKI risk stratification of the critically ill patients with sepsis is critical. Although the prevention of AKI in critically ill patients has formed a certain consensus, instances, where a recommendation cannot or should not be followed for an individual patient, have been reported [11]. Rather than using biomarkers or clinical risk factors alone, derived based on large population or cohort data, the nomogram provides a more individualized admonition for risk information to septic patients. For example, the European Society of Intensive Care Medicine suggests a mandatory review of all medications with cessation of nephrotoxic ones in addition to the treatment measures [11]. However, many aspects may affect the treatment strategy of septic patients and the lesions changed quickly, clinicians depend on their clinical experience. Clinicians may be more accurate in selecting treatment strategies for a higher probability of benefiting from treatments, using a nomogram with clinical factors.

The most important and final argument for the use of the nomogram is based on individual needs for additional investigation or care [22]. Although is have good prediction performance, discrimination and calibration, the nomogram cannot capture the clinical consequences of miscalibration or a particular level of discrimination. The DCA is a valuable decision-making tool when different means are compared with their clinical value [34–36]. Therefore, to justify its clinical utility, DCA was applied to unravel the usefulness of nomogram 2 decisions. This novel method provides insights into clinical consequences based on threshold probabilities, where a net benefit is derived [34]. The decision curve revealed that nomogram 2 caused a positive net benefit with a threshold probability of between 15 and 80 %. For example, if the threshold probability of a patient is 40 %, the net benefit would be 15 % when nomogram 2 is used to predict AKI, hence more benefit than either the treat-none or the treat-all scheme.

Despite these promising findings, this paper has some limitations. First, the nomogram was retrospectively constructed and new biomarkers were not included in the analysis, potentially reducing the performance of the model. Secondly, the urine standard was not used in the diagnosis of AKI since urine volume data may be unreliable due to the use of diuretics. This may reduce the overall incidence rate of AKI. Thirdly, the missing data were settled with median imputation and multiple imputation techniques, potentially decreasing the accuracy and C-index of the final model. Nonetheless, data for these predictors can easily be obtained in the intensive care units and not an obstacle in implementing the nomogram. Furthermore, these disadvantages are natural in any retrospective studies, and population-based research thereby increasing concerns on the stability of the results. Nevertheless, this nomogram may somewhat help clinicians make reasonable risk judgments and treatment strategies in the absence of high-quality SA-AKI prediction tools.

## Conclusions

This study developed and verified an AKI risk prediction nomogram applied to critically ill patients with sepsis, which may partially help clinicians make reasonable risk decisions and treatment strategies. Nonetheless, further verification using external data is essential to enhance the applicability of this nomogram in clinical practice.

## Data Availability

The datasets used and/or analysed during the current study are available from the corresponding author upon reasonable request.

## Abbreviations

AKI: Acute kidney injury
SA-AKI: Sepsis associated-AKI
MIMIC-III: Medical Information Mart for Intensive Care III
BMI: Body mass index
SCr: Serum creatinine
LOS in ICU: Length of stay in intensive care unit
SIRS: Systemic inflammatory score
COPD: Chronic obstructive pulmonary disease
CKD: Chronic kidney disease
